# Effect of high-intensity laser therapy versus shockwave therapy on selected outcome measures in osteoporotic long-term hemiparetic patients: a randomized control trial

**DOI:** 10.1186/s13018-023-04141-5

**Published:** 2023-09-02

**Authors:** Tamer I. Abo Elyazed, Islam M. Al-Azab, Ahmed Abd El-Moneim Abd El-Hakim, Sabah Mohamed Elkady, Rabab Mohamed Monged Afifi, Hany Ezzat Obaya

**Affiliations:** 1 Department of Physical Therapy for Internal Medicine, Faculty of Physical Therapy, Beni-Suef University, Beni Suef, Egypt; 2 Department of Physical Therapy for Neuromuscular Disorders and Its Surgery, Faculty of Physical Therapy, Cairo University, Giza, Egypt; 3 Department of Physical Therapy for Neuromuscular Disorders and its Surgery, Faculty of Physical Therapy, October 6th University, Giza, Egypt; 4 Department of Basic Sciences, Faculty of Physical Therapy, Beni-Suef University, Beni Suef, Egypt; 5 Department of Basic Science, Faculty of Physical Therapy, Cairo University, Giza, Egypt; 6 Department of Orthopedic and Sport Injuries, October 6th University, Giza, Egypt; 7 Department of Physical Therapy for Cardiovascular/Respiratory Disorder and Geriatrics, Faculty of Physical Therapy, Cairo University, Giza, Egypt

**Keywords:** Hemiplegia, High-intensity laser therapy, Osteoporosis, Shock wave therapy

## Abstract

**Background:**

This study aimed to compare the effects of high-intensity laser therapy (HILT) and extracorporeal shock wave therapy (ESWT) in treating consequences of osteoporosis in hemiparetic patients.

**Methods:**

A randomized controlled trial was conducted on hemiplegic patients with osteoporosis. They were randomly classified into three equal groups (*n* = 40 in each group). The control group received medication and traditional physiotherapy programs for stroke patients. The high-intensity laser (HIL) group received the same intervention as the control group in addition to high-intensity laser therapy. The shock wave (SW) group received the same intervention as the control group in addition to shock wave therapy. The three groups received an intervention that lasted 3 sessions/week for 12 weeks). All groups were assessed before and after therapy for the degree of pain, fall risk, and quality of life.

**Results:**

A statistically significant difference (*p* < 0.05) was found concerning VAS, which had a significant difference in favor of HILT and ESWT groups compared to the control group; however, no significant difference was determined between HIL and SW groups. Regarding the overall stability index, SFBBS, and QUALEFFO-41, there was a significant difference in favor of HIL and SW groups compared to the control group, and a significant difference was found in HIL when compared to SW.

**Conclusion:**

The current study indicates that the combined traditional physical therapy and HILT and ESWT have clinical significance in improving osteoporotic long-term hemiparetic patients with more favor to HILT.

*Trial registration:* The study was registered as a clinical trial at ClinicalTrial.gov ID (NCT05616611).

## Introduction

Stroke is one of the most challenging medical conditions in the world. Stroke-related motor disability is leading to miscellaneous complications. Osteoporosis and consequent fractures are well known as stoke-related complications [1]. Osteoporosis considerably increases fracture risk and causes a heavy financial burden on healthcare systems. It is characterized by decreased bone cell mass and tensile strength, and distortion of the skeletal microarchitecture [2].

Hemiparetic patients are predisposed to fractures due to considerable loss of bone mass on the affected side; also, the possibility of falling could be increased [3]. Painful hips and back due to osteoporosis in hemiparetic patients have been explored in many reviews as a disability aggravating factor adding more disability to stroke patients and more significant loss of daily activities [4, 5]. Quality of life is negatively influenced by fragility fractures which are common in the elderly [6]. A significant health care challenges were to find proper management of osteoporosis, reduce the occurrence of osteoporosis and associated consequences and fractures in stroke survivors [2, 7].

Bisphosphonates were used as first-line therapy. However, alendronate and denosumab were found to be effective in managing patients with corticosteroids induced osteoporosis, on the other side denosumab approved in enhancing bone mineral density of the axial spine and hips and reducing incidence of fractures in women with postmenopausal osteoporosis [8–10].

Changing one’s diet, taking vitamin D supplements, calcium, and bisphosphonates, which prevent osteoclastic bone resorption, considered as part of traditional therapy [11]. Although these drugs are valuable, they all have side effects and complications that make long-term use challenging [12, 13].

High-intensity laser therapy (HILT) and extracorporeal shock wave therapy (ESWT) are promising modalities which affect bone mass as an alternative therapy. HILT is a relatively new technology. The high-power pulsed Nd: YAG laser has a high peak power and can target deep tissues, contrary to traditional lasers [14].

Along with thermal processes, HILT causes chemical, mechanical, and other changes [15]. Profibrinolytic effects may be brought on by the mechanical and thermal impacts of HILT beams. HILT quickly eases discomfort and inflammation [16]. It does this by inducing deeper tissue photochemical and photothermic effects using a waveform of short duration and regular amplitude peaks [17].

A quick, brief-duration acoustic wave called an extracorporeal shock wave can carry energy and penetrate tissue. This would be a mechanical stimulation that biologically impacts living tissue [18–20]. Through one of the following processes, extracorporeal shock wave treatment (ESWT) may successfully stimulate the production of new bone. ESWT will first increase the expression of growth factors [21–23]. Then, ESWT can yield more osteoblasts from stem cells [21, 24]. Third, ESWT is also associated with neovascularization, which increases local blood flow and, as a result, improves cell metabolism, speeds up the development of new bone, and reduces inflammation [25–27].

A prolonged period is consumed for any intervention used for the treatment of osteoporosis. Therefore, different modalities will be enrolled for rapid relief of osteoporotic patients’ issues such as pain, balance, and poor quality of life. Some researches approved the efficiency of HILT and ESWT on decreasing pain in musculoskeletal systems and improving dynamic and static balance which in turn could decrease falling in elderly and hemiparetic patients. The scarcity of studies concentrating on osteoporosis among hemiparetic patients makes it challenging to develop effective intervention programs that meet their specific issues. To the best of our knowledge, no study has compared the effects of HILT and ESWT in the treatment of osteoporosis consequences such as pain, balance and falling in hemiparetic patients. This study aimed to determine the outcomes of HILT and ESWT in treating osteoporosis consequences in individuals with hemiparesis.

## Materials and methods

### Study design

A randomized controlled trial was carried out at the Faculty of Physical Therapy Outpatient Clinic, Cairo University, between October 2021 and March 2022. The ethical committee of the Physical Therapy Faculty at Cairo University authorized this work (No: P.T.REC/012/003393). The study was registered as a clinical trial at ClinicalTrial.gov ID (NCT05616611).

### Participants

One hundred and forty hemiplegic patients with osteoporosis of both sexes were chosen randomly. Hemiparetic chronicity ranges from eight to ten years. Their age ranged from 60 to 70 years old. All participants were diagnosed by dual-energy X-ray absorptiometry (DEXA) as osteoporosis or osteopenia, where T-score was − 1.5 or less. All patients were recruited from Kasr Al-Ainy Hospital, Cairo University. The patients were informed about the aim of the study. All the patients signed a consent form for the participation agreement.

### Exclusion criteria

BMI of more than 30 or less than 18, advanced musculoskeletal disorders, rheumatoid arthritis, skin diseases, long-term steroids therapy, or any drug affecting bones.

### Randomization

**Fig. 1:**
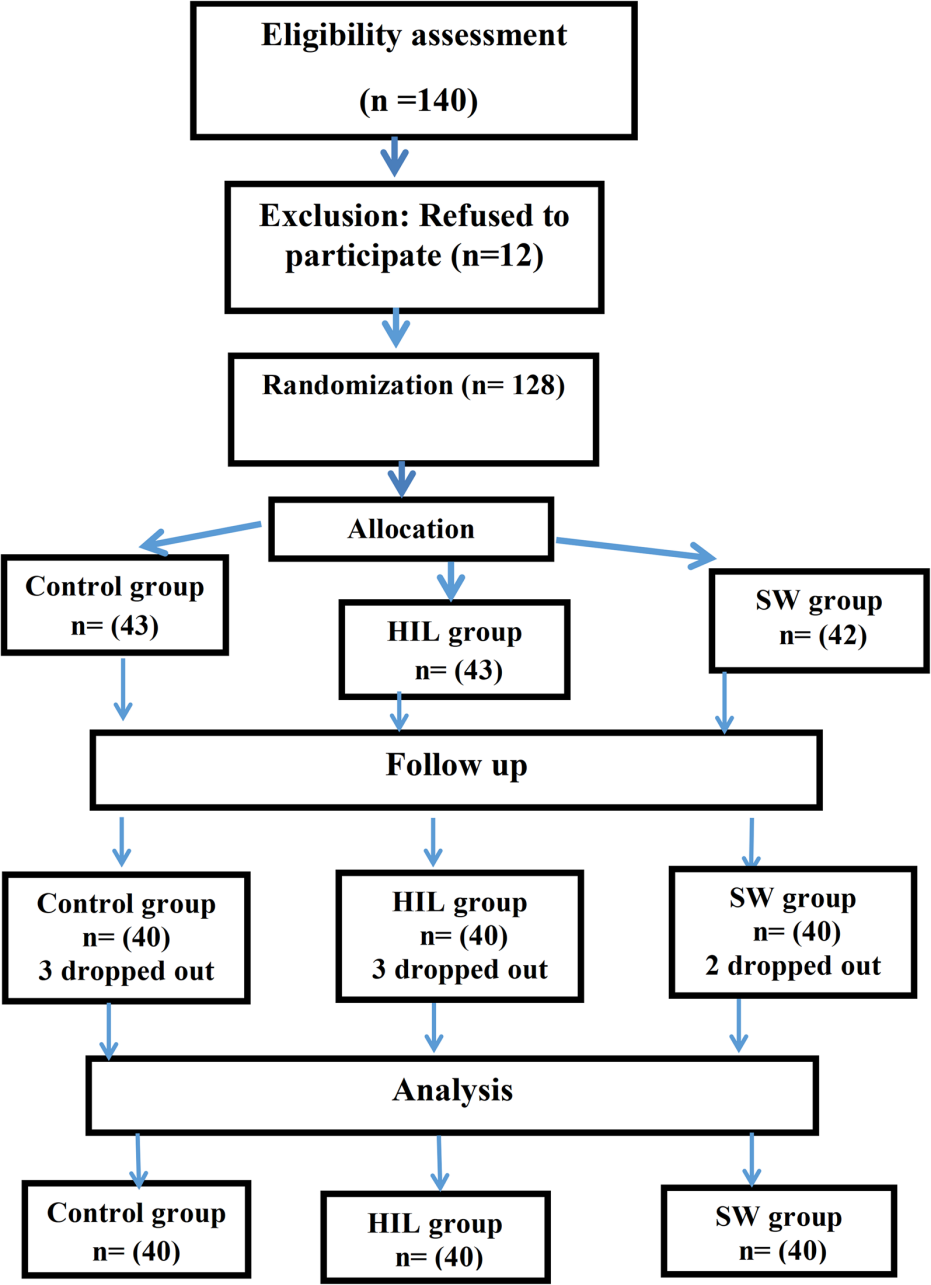
Flowchart of the participants

One hundred and forty hemiplegic patients with osteoporosis were evaluated for eligibility; 12 patients disagreed to participate in this study. Therefore, one hundred and twenty-eight were randomly divided into three groups of equal size, using random allocation software (GraphPad Software Inc.) to minimize selection bias**.** Eight patients dropped out from post-treatment assessment. A diagram of the patient’s flow and randomization is shown in Fig. 1.

The patients were sorted into three groups randomly through the GraphPad QuickCalcs website (GraphPad Software Inc.) (*n* = 40 in each group after follow-up). The control group received medication and a traditional physiotherapy program for stroke patients (strengthening, stretching, and balance exercise). The high-intensity laser (HIL) group received the same intervention as the control group and high-intensity laser therapy. The intervention of the shock wave (SW) group was the same as that of the control group in addition to shock wave therapy. The groups interventions lasted 3 sessions/week for three months).

### Outcomes measurements

1. Visual analog scale (VAS): a measurement tool used to evaluate patient discomfort as his/her experience to a specific degree**.** All patients in each group were evaluated by VAS before and after the treatment interventions.
2. Fall risk assessment: This was performed by two methods: Overall stability index which was obtained by (Biodex Balance Master, USA) the second method was the Short Form of Berg Balance Scale (SFBBS), commonly used to evaluate dynamic balance in clinical research by assessing a patient’s ability to perform seven transitional movement items [28]**.** Both methods were carried out for the patients in the three groups before and after treatment interventions.
3. Quality of life assessment: It was performed using the Quality of Life Questionnaire of the European Foundation for Osteoporosis (QUALEFFO-41). This is the most frequently used for patients with osteoporosis. QUALEFFO-41 covers not only patients’ aspects of pain or physical functions but also aspects of social and cognitive functions [29]**.** A valid and reliable Arabic version of QUALEFFO-41 was introduced to all participants pre- and post-treatments interventions.

### Therapeutic procedures

#### High-intensity laser therapy (HILT)

HIL was produced by ASA S.r.l.—Italy EN 60825, Nd: YAG laser, with an average power of 10.5 W and wavelength of 1064 nm. All the patients in the HIL group received three HILT sessions per week for three months. The lower back and both hips were scanned by the device by 3000 J; the average fluency was 15–10 J cm^2^. Each area received approximately 10 min of HILT [30]**.**

### Shock wave therapy

Focused ESWT with 2000 pulses and energy of 0.12 mJ mm^2^ was applied to the lumbar region and both hips [31]. All the patients in the SW group received ESWT three sessions per week for three months.

### Power analysis

The sample size was determined using G*Power (version 3.1.9.2; Germany) [F tests-MANOVA: Repeated measures, within-between interaction], using an effect size of 0.135, a 95% power analysis, and a two-sided 5% significant level. As a result, the total estimated sample size for patients was 120 patients.

### Statistical analysis

The statistical analyses were calculated by SPSS version 20. The mean and SD were used to describe the study results. Paired t test was used to compare each group pre and post-intervention. ANOVA was used to compare the three groups’ clinical features and outcome measures before and after the intervention. Qualitative data were analyzed by chi-square test. P value of ≤ 0.05 was considered statistically significant.

## Results

A comparison of participants’ demographic data (age, height, weight and sex) and illness duration between the groups (Table 1) using the ANOVA test and chi-square test revealed a non-significant difference.

**Table 1:** Comparison of participants’ demographic data and illness duration between groups

Item	HIL group	Shock W. group	Control group	*p*
	Mean ± SD	Mean ± SD	Mean ± SD	
1—Age (years)	61.70 ± 4.44	62.35 ± 4.66	61.15 ± 4.23	0.69
2—Height (cm)	169.05 ± 4.56	169.60 ± 4.39	169.70 ± 3.54	0.443
3—Weight (kg)	85.55 ± 4.45	85.70 ± 4.73	84.80 ± 4.37	0.714
4—Duration of illness/(years)	8.45 ± 1.23	8.50 ± 1.19	8.25 ± 1.16	0.793
5—Sex	No. (%)	No. (%)	No. (%)	
Male	32(80%)	33(82.5%)	31(77.5%)	0.9778
Female	8(20%)	7(17.5%)	9(22.5%)	

HIL, High-intensity laser, Shock w, shock wave; SD, standard deviation

*p* > 0.05 = Non-significant; *p* ≤ 0.05 = significant*; *p* ≤ 0.01 = highly significant**

Comparison of variables means values between pre and post-treatment for each group using t test and between groups pre- and post-treatment using ANOVA (Table 2), where P value considered significant at *p* < 0.05 showed that there was no significant difference between groups for all variables pre-treatments. At the same time, there was a significant difference between each group pre and post-treatment for all variables except VAS, overall stability index, and QUALEFFO-41 of the control group. In contrast, the comparison of VAS, overall stability index, SFBBS, and QUALEFFO-41 between the three groups post-treatment revealed significant differences (*p* < 0.05).

**Table 2:** Comparisons of the mean values for intra- and inter-groups pre and post-treatment

		Control Group (mean ± SD) (n = 40)	HIL Group (mean ± SD) (n = 40)	SW. Group (mean ± SD) (n = 40)	F	*p* value*
VAS	Pre-treatment	7.26 ± .98	7.2 ± 1.4	7.06 ± 1.2	0.273	0.762^NS^
	Post-treatment	6.96 ± 1.3	4.66 ± 1.4	4.03 ± 0.93	45.524	0.000^S^
	*p* value**	0.071^NS^	0.000^S^	0.000^S^		
Overall Stability Index	Pre-treatment	3.17 ± 0.37	3.17 ± 0.34	3.15 ± 0.36	0.014	0.987^NS^
	Post-treatment	3.15 ± 0.40	1.85 ± 0.42	2.88 ± 0.38	85.76	0.000^S^
	*p* value**	0.784^NS^	0.000^S^	0.000^S^		
SFBBS	Pre-treatment	21.30 ± 1.6	21.33 ± 1.7	21.83 ± 1.38	0.866	0.424^NS^
	Post-treatment	21.86 ± 1.6	24.46 ± 1.04	23.26 ± 2.70	13.863	0.000^S^
	*p* value**	0.000^S^	0.000^S^	0.001^S^		
QUALEFFO-41	Pre-treatment	107.5 ± 1.3	104.5 ± 0.2	102.5 ± 1.9	0.881	0.80^NS^
	Post-treatment	117.5 ± 1.4	170. ± 1.6	137.5 ± 0.9	93.4	0.000^S^
	*p* value**	0.330^NS^	0.000^S^	0.000^S^		

HIL, High-intensity laser; SW, shock wave; VAS, visual analog scale; SFBBS, Short Form of Berg Balance Scale; and QUALEFFO-41, Quality of Life Questionnaire of the European Foundation for Osteoporosis

* Inter-groups comparison; ** intra-group comparison of the results pre- and post-treatment

^NS^*p* > 0.05 = non-significant, ^S^*p* < 0.05 = significant, *p* = probability

The postdoc test was used to assess any difference between the three groups’ post-treatment mean values of the measured variables **(**Table 3). This showed that there was a significant difference in favor of HIL and SW groups concerning VAS compared to the control group. In contrast, no significant difference was determined between HIL and SW groups. Considering the overall stability index, SFBBS, and QUALEFFO-41, there was a significant difference in favor of HIL and SW groups compared to the control group, and there was a significant difference between HIL when compared to SW.

**Table 3:** Post hoc test inter-groups for VAS, overall stability index, SFBBS, and QUALEFFO-41

Variables	Groups comparison	Mean difference	*p* value
VAS	Con. versus HIL*	2.30000	0.000^S^
	Con v versus SW*	2.93333	0.000^S^
	HIL versus SW	0.63333	0.129^NS^
Overall stability index	Con. versus HIL*	1.29667	0.000^S^
	Con versus SW*	0.27333	0.028^S^
	HIL* versus SW	1.02333	0.000^S^
SFBBS	Con. versus HIL*	2.60000	0.000^S^
	Con versus SW*	1.40000	0.016^S^
	HIL* versus SW	1.20000	0.045^S^
QUALEFFO-41	Con. versus HIL*	54.175	0.000^S^
	Con versus SW *	20.825	0.049^S^
	HIL* versus SW	26.66	0.001^S^

Con., Control group; HIL, High-intensity laser group; S.W., shock wave group; VAS, visual analog scale; SFBBS, Short Form of Berg Balance Scale; and QUALEFFO-41, Quality of Life Questionnaire of the European Foundation for Osteoporosis

* Significant group

^NS^*p* > 0.05 = non-significant, ^S^*p* < 0.05 = significant, *p* = probability

## Discussion

In order to assess the effectiveness of HILT and ESWT in treating osteoporotic long-term hemiparetic patients, this study was carried out. Outcome measures were visual analog scale (VAS), fall risk which was assessed using two different techniques: the overall stability index (Biodex Balance Master, USA) and the Short Form of Berg Balance Scale (SFBBS).

A valid and reliable quality of life questionnaire to measure health-related quality of life was carried out by the Quality of Life Questionnaire of the European Foundation for Osteoporosis (QUALEFFO-41).

The primary conclusion is that combining HILT and ESWT with traditional physical therapy is more effective in increasing the overall stability index, (SFBBS), quality of life questionnaire, and lowering the VAS scores following a 12-week physical therapy alone. The results showed that although both HILT and ESWT were effective in improving the overall stability index (SFBBS) (QUALEFFO-41) and lowering the VAS ratings after receiving therapy for 12 weeks than traditional physical therapy alone in osteoporotic long-term hemiparetic patients, HILT was more effective than ESWT except for VAS.

Improving the overall stability index, (SFBBS), quality of life questionnaire, and VAS scores after treatment with the modalities used in this study was reflected on the stroke patients, their ADL scores, and activity as observed in their quality of life questionnaire.

The best course of treatment for osteoporotic long-term hemiparetic patients was high-intensity laser therapy (HILT) in conjunction with conventional physical therapy. Patients with the osteoporotic bone are currently treated with low-intensity laser therapy. It is a valuable physical treatment technique for enhancing range of motion [32, 33] and reducing chronic low back pain radiculopathy, functional impairment, and pain levels [34]. A recent development in HILT has been using pulsed Nd: YAG laser treatment for various ailments. Applications for HILT included comfort from pain [34]. It was used to treat the arthritic pain of multiple sources [35–38]

Shortly, HILT and ultrasonic wave therapy for patients with low back discomfort were compared by Fiore et al. Participants in the study underwent HILT for three weeks in a row. They demonstrated a substantial reduction in pain that was greater than ultrasound therapy [39]. According to the features of laser wavelength and coherence, laser treatment was thought to change tissue function typically [40].

Since human skin lacks enough endogenous chromophores to absorb laser wavelength (1064 nm) effectively, the pulsed Nd: YAG laser operates in a therapeutic model that enables it to be absorbed and propagated by tissue more rapidly [41]. The scattering effect, which causes light to diffuse in all directions during tissue-level absorption, boosts the mitochondrial oxidative reaction and, as a result, enhances the generation of adenosine triphosphate (ATP), DNA, adenosine triphosphate (ATP), and RNA. This phenomenon was known as photobiology [41].

Thermal build-up happens when the Nd: YAG laser is utilized continuously. HILT employed a specific waveform. 3 kW of power was reached, with frequent peaks of high amplitude for a minimal period and ultra-short duty cycle to quickly initiate photochemical and photothermal effects while reducing the thermal build-up in deep tissues [41]. These characteristics lead to treating deeper tissues and structures due to increased radiation production in the treated tissues with no histological changes.

By varying the pulse frequency and strength, the photothermal effect might be managed for patient comfort and safety [42, 43]. Numerous musculoskeletal problems have been successfully treated using pulsed Nd: YAG lasers, which were also thought to counter inflammation and enhance tissue repair [42].

The sedation impact of HILT depends on various modes of action, such as its ability to decrease the velocity of pain stimuli and boost the body’s creation of morphine-like compounds [44]. Additionally, it could immediately impact nerve cells, which might speed the recovery from a conduction block or stop the transmission of A- and C-fibers [44].

In a study involving rabbits, it was found that the application of ESWT enhanced neovascularization and the expression of osteogenic and angiogenic growth factors at the bone junction of the Achilles tendon, including proliferating cell nuclear antigen (PCNA), endothelial nitric oxide synthase (eNOS), bone morphogenic protein-2 (BMP-2), and vessel endothelial growth factor (VEGF2 [45, 46]. Following the administration of ESWT, the growth in neo-vessels started to increase one week later, peaked after four weeks, and continued for the next twelve weeks. After reaching their highest levels after 3 months, eNOS, VEGF, and BMP-2 gradually decreased to baseline after 12 weeks. Following the application of ESWT, the change in PCNA began to increase one week later, peaking after 12 weeks. The findings suggest that ESWT induces neovascularization to increase starting from one week after treatment; this effect lasted more than 12 weeks after therapy.

This was further corroborated by the fact that PCNA was still elevated 12 weeks after treatment, although eNOS, VEGF, and BMP-2 effects had mostly reverted to their pre-treated levels. The biological reactions have been demonstrated in the literature for the first time. Several investigations, including non-union fractures, showed comparable findings [46, 47].

ESWT was used to treat individuals who had femoral head osteonecrosis. In particular research, the biological basis for ESWT’s use in treating osteonecrosis of the hip joint was investigated [48, 49]. Compared to hips without ESWT before hip replacement, those treated with ESWT had more viable bone tissue, greater cell concentrations, and increased cell activities such as phagocytosis. When compared to patients without ESWT before surgery, molecular expression analyses revealed significant increases in vWF, VEGF, CD 31, Wnt3, and PCNA, and significant reductions in VCAM and Dickkopf-1 (DKK-1).

In a different research, six individuals with osteonecrosis had their bone marrow stromal cells (BMSCs) removed from the femur [50]. In the shockwave group, there were noticeable increases in the levels of VEGF, cell proliferation, alkaline phosphatase, runt-related transcription factor 2 (RUNX2), osteoclast, and BMP-2. These findings approved that ESWT increases the osteogenic and angiogenic effects of BMSCs in hips with osteonecrosis through the nitric oxide route [48, 50].

Extraneous factors may be corroborated to current study limitation which may have interfered with our results, such as variations in life style between patients as activity level, and psychological factor of the participants during the period of application of the study. Regarding to osteoporosis duration, it was difficult to be declared from the patients due to economic and social reasons. Analysis of gender differences on different responses of High-Intensity Laser Therapy and Shockwave Therapy on osteoporotic long-term hemiparetic patients could be a recommended future research.

## Conclusion

According to the current study, combining standard physical therapy with HILT and ESWT has been clinically significant in treating osteoporotic long-term hemiparetic patients, favoring HILT more than ESWT. The visual analog scale (VAS), the Short Form of Berg Balance Scale (SFBBS), and the Quality of Life Questionnaire of the European Foundation for Osteoporosis (QUALEFFO-41) were used to assess clinical improvement in the current study. Fall risk was also assessed by the overall stability index and the Quality of Life Questionnaire of the European Foundation for Osteoporosis. These results may require further investigation using measures of bone mineral density (DEXA).

## Data Availability

The data associated with the paper are not publicly available but are available from the corresponding author on reasonable request.

## Abbreviations

HILT: High-intensity laser therapy
ESWT: Extracorporeal shock wave therapy
QUALEFFO-41: Quality of Life Questionnaire of the European Foundation for Osteoporosis
SFBBS: Short Form of Berg Balance Scale
VAS: Visual analog scale
